# In Situ Construction of Efficient Interface Layer with Lithiophilic Nanoseeds toward Dendrite‐Free and Low N/P Ratio Li Metal Batteries

**DOI:** 10.1002/advs.202104391

**Published:** 2022-01-25

**Authors:** Lingli Luo, Shuixin Xia, Xun Zhang, Junhe Yang, Shiyou Zheng

**Affiliations:** ^1^ School of Materials Science & Engineering University of Shanghai for Science and Technology Shanghai 200093 China; ^2^ Ganjiang Innovation Academy Chinese Academy of Sciences Ganzhou 341000 China

**Keywords:** energy storage, lean electrolyte, Li metal anode, low N/P

## Abstract

Li metal is considered as one of the most promising candidates for constructing advanced high‐energy energy storage due to its ultrahigh theoretical capacity and lowest electrochemical potential. However, its practical commercialization is seriously hindered by the challenges of Li dendrite growth, low Coulombic efficiency, and huge volumetric variation. Herein, an efficient in situ generated Li_2_S‐rich interface layer joint with preplanted Sb nano active sites in hosted Li metal anode is easily achieved with the nanosized Sb_2_S_3_ decorated carbonaceous network. The yielded CC@Sb_2_S_3_@Li anode demonstrates uniform Li deposition, high Coulombic efficiency, and alleviated volumetric variation. Therefore, the Li symmetric cells show ultralong lifespan stable cycling over 3200 cycles with a very low voltage hysteresis (≈18 mV) at 5 mA cm^−2^. Impressively, the Li|LiFePO_4_ full cell delivers an exceptionally prolonged cycling over 180 cycles with a superior capacity retention as high as ≈90% even under the harsh condition of an extremely low negative to positive capacity ratio of ≈0.44 with lean electrolyte (4.4 µL mAh^−1^). Moreover, the Li|LiNi_0.5_Co_0.2_Mn_0.3_O_2_ full cell also maintains an excellent cycling performance under the more realistic harsh conditions. This work provides a new avenue and significant step paving the Li metal toward the practical application.

## Graphical abstract

Sb_2_S_3_ decorated carbonaceous 3D architecture is rationally exploited to stabilize Li metal. An efficient Li_2_S‐rich interface layer and lithiophilic Sb nucleation sites are in situ constructed. The Li symmetric cells demonstrate an ultralong lifespan stable cycling and the full cells deliver extraordinarily prolonged cycling performance even under the harsh conditions of extremely low negative/positive capacity ratio and lean electrolyte.

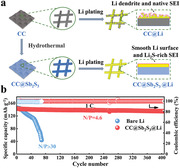

## 1 Introduction

To meet the growing demand for high energy density applied in electric transportation and smart grids, advanced high capacity electrodes are urgent to be developed.^[^ ^1^, ^2^ ^]^ Li metal, known as the “Holy Grail” of anode materials, is possessed with the ultrahigh theoretical capacity (3860 mAh g^−1^, ≈10 times of conventional graphite), the lowest electrochemical potential (−3.04 V vs standard hydrogen electrode), and low density (0.534 g cm^−3^).^[^ ^3^, ^4^ ^]^ The realization of Li metal substitutes for the conventional graphite can promote a big leap in the overall cell energy density.^[^ ^5^, ^6^, ^7^ ^]^ Nevertheless, its commercialization has severely been hindered by the notorious growth of Li dendrites, inducing cell short circuit and even serious fire.^[^ ^8^, ^9^ ^]^ Also, the infinite volume change during the charging/discharging process is still another crucial issue to be solved, which will cause serious interfacial contact failure.^[^ ^10^, ^11^ ^]^

Tremendous strategies have been raised to tackle with the above thorny issues, including electrolyte additives,^[^ ^12^, ^13^, ^14^ ^]^ solid‐state electrolytes,^[^ ^15^, ^16^ ^]^ artificial solid electrolyte interlayers (SEI).^[^ ^17^, ^18^, ^19^ ^]^ Despite the electrochemical performance of Li metal can be enhanced to some extent, the dramatic volume change caused by the “hostless” nature of Li is also a seriously encountered challenge.^[^ ^20^ ^]^ Particularly, the native SEI on Li metal surface is susceptible to be collapse enduring the huge strain change during the repeated Li plating/stripping cycles, which finally aggravates notorious dendritic Li growth and low Coulombic efficiency (CE). Thus, it is extremely imperative to simultaneously achieve both the robust SEI layer and volumetric stable metallic Li architecture to guarantee the high performance of Li metal anode.

Various 3D structured materials have been raised to accommodate Li metal. Particularly, carbon‐based skeletons are considered as ideal candidate materials for Li metal host due to its low cost and low density.^[^ ^21^, ^22^ ^]^ Currently, new emerging 3D carbon materials, such as graphene and its derivatives,^[^ ^23^ ^]^ carbon nanofibers (CNF),^[^ ^24^ ^]^ carbonized MOF,^[^ ^25^ ^]^ hollow carbon spheres,^[^ ^26^ ^]^ and porous carbon^[^ ^27^ ^]^ etc. have been successfully applied as lithium metal scaffolds. However, the complicated preparation procedure has seriously restricted its large‐scale production and utilization. Meanwhile, to achieve the full utilization of the carbonaceous host, the mismatch of Li metal with the host matrix should also be solved. And additional lithophilic sites, such as metals,^[^ ^28^, ^29^ ^]^ oxides,^[^ ^30^ ^]^ and inorganic N,F active sites^[^ ^22^, ^27^ ^]^ are usually indispensable. Impressively, metal sulfides demonstrate promising application prospects in lithium batteries owing to its excellent electronic conductivity.^[^ ^31^, ^32^, ^33^ ^]^ Li_2_S and Li‐based alloy can be in situ formed when the metal sulfide reacts with lithium, which is also conducive to the homogeneous Li nucleation and growth.^[^ ^34^, ^35^ ^]^

Here, a robust lithiophilic carbonaceous scaffold enabled by nanosized Sb_2_S_3_ is synthesized via a facile one‐step hydrothermal synthesis. During the lithiation, highly lithiophilic nano Sb sites and Li_2_S‐rich SEI can be in situ generated, which could facilitate the uniform Li^+^ flux and guide the uniform Li deposition. And the 3D carbonaceous architecture can effectively reduce the local current density and alleviate the huge volume change during the repeated Li plating/stripping. Consequently, the Li symmetric cells display ultralong lifetime cycling stability with low polarization voltage. When matched with high‐loading LiFePO_4_ (LFP) cathode (≈13.36 mg cm^−2^), the Li|LFP cell exhibits prolonged lifespan cycling stability with a superior high capacity retention of ≈94% at 1 C. The Li|LFP cell still can demonstrate an impressive long‐term and extraordinary cycling stability with an excellent high capacity retention of ≈90% over 180 cycles under the harsh condition (the negative to positive capacity ratio of ≈0.44; the electrolyte to positive capacity ratio of ≈4.4 µL mAh^−1^). Moreover, the Li|LiNi_0.5_Co_0.2_Mn_0.3_O_2_ (Li|NCM523) full cell also maintains an excellent cycling performance under the more realistic conditions. This work demonstrates a significant breakthrough stimulating the Li metal anode toward practical usage.

## 2 Results and Discussion

**Figure 1:**
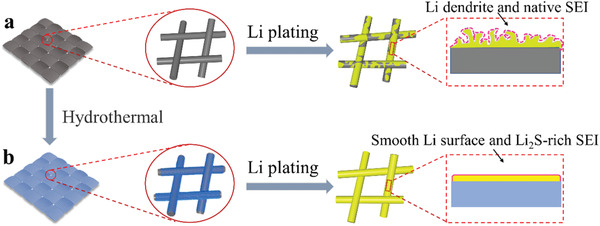
Schematic illustration of Li deposition on different substrates. a) bare CC and b) CC@Sb_2_S_3._

**Figure** **1** shows the schematic illustration of lithium deposition on different substrates. For bare carbon cloth (CC), lithium ions are prone to distribute inhomogeneously due to highly lithiophobic nature of the substrate, resulting in uneven Li deposition and the dendritic Li growth. In contrast, when CC enabled by nanosized Sb_2_S_3_, Li_2_S‐rich SEI layer, and lithiophilic Sb active sites can be in situ generated via the lithiation reaction. The Li_2_S‐rich SEI joint with the lithiophilic Sb active sites can homogenize Li^+^ flux contributing to the uniform Li nucleation and the conformal growth. Moreover, the huge volume change can be mitigated owing to the 3D carbonaceous architecture. Consequently, the achieved hosted Li delivers excellent electrochemical performance.

**Figure 2:**
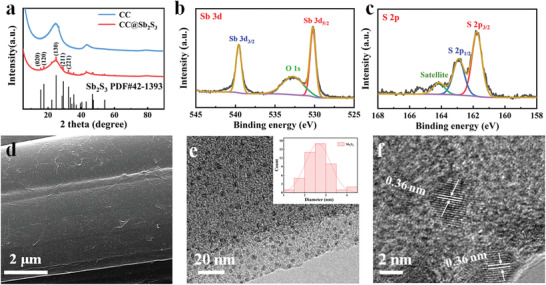
Phase structure and morphology characterizations of CC@Sb_2_S_3_. a) XRD patterns of CC and CC@Sb_2_S_3_. High‐resolution XPS spectras of b) Sb 3d and c) S 2p of CC@Sb_2_S_3_. d) SEM image and e) TEM images of CC@Sb_2_S_3_ (the inset is the particle size distribution of Sb_2_S_3_ on CC@Sb_2_S_3_). f) The high‐resolution TEM image of CC@Sb_2_S_3_.

Sb_2_S_3_ nanoclusters decorated CC (denoted as CC@Sb_2_S_3_) is prepared by a simple one‐step hydrothermal method. X‐ray diffraction (XRD) characterization is conducted to determine the physical structure changes during the hydrothermal treatment (**Figure** **2**a). For bare CC, two broad peaks are detected at 25° and 43°, which are the characteristic diffractions of amorphous carbon. When Sb_2_S_3_ are loaded onto CC, characteristic peaks centered at 15.6, 17.5, 24.9, 29.2 and 32.4° can be detected in addition to the characteristic diffraction of the carbon matrix, which can be assigned to the (020), (120), (130), (211) and (221) lattice planes of Sb_2_S_3_ (JCPDS no. 42‐1393), respectively. X‐ray photoelectron spectroscopy (XPS) test is also carried out to identify the electronic state of elements. Figure 2b shows the high‐resolution spectra of Sb 3d, which can be deconvoluted into the two peaks located at 530.8 and 539.6 eV designated to Sb 3d_5/2_ and Sb 3d_3/2_. The XPS results reveal the existence of Sb^3+^ in Sb_2_S_3_.^[^ ^36^, ^37^ ^]^ At the same time, it is noted that the peak at 533 eV can be designated to the oxygen absorbed on surface of Sb_2_S_3_. For the S 2p spectrum (Figure 2c), peaks centered at 161.5 eV (S 2p_3/2_) and 162.6 eV (S 2p_1/2_) could be testified, which are corresponded to the signals of S^2−^ in Sb_2_S_3_. Also, shake‐up satellite peak of S located at high binding energy is detected. The Sb_2_S_3_ atomic content calculated from the XPS survey spectrum is ≈0.53% (Figure S1, Supporting Information). The energy dispersive spectrometer (EDS) image of CC@Sb_2_S_3_ shows only the peaks of C, Sb, and S elements (Figure S2, Supporting Information). Scanning electron microscope (SEM) characterization is conducted to track the Li deposition behavior with different Li plating capacity. As shown in Figure S3 (Supporting Information), bare CC is composed of micron‐sized carbon fibers interleaving together with each other, which shows a smooth surface with an average diameter of ≈5.4 µm. After hydrothermal synthesis, nanosized Sb_2_S_3_ particles can be detected uniformly loaded on the surface of carbon fibers without obvious aggregation (Figure 2d). Transmission electron microscope (TEM) is used to further identify the nanoparticles. Figure 2e demonstrates the TEM image of CC@Sb_2_S_3_. The corresponding particle size distribution of the Sb_2_S_3_ clearly shows that the nanoparticle size is less than 10 nm. The high‐resolution TEM image reveals an obvious lattice spacing of ≈0.36 nm in well accordance with the (130) crystal plane of Sb_2_S_3_ (Figure 2f). The resulted CC@Sb_2_S_3_ scaffold was directly used as the scaffold to host Li metal anode via the electroplating method. After Li deposition, no other diffraction peaks can be observed except for that of Li metal and carbon host (Figure S4, Supporting Information), which could be ascribed to the quite low loading of Sb_2_S_3_ on CC.

**Figure 3:**
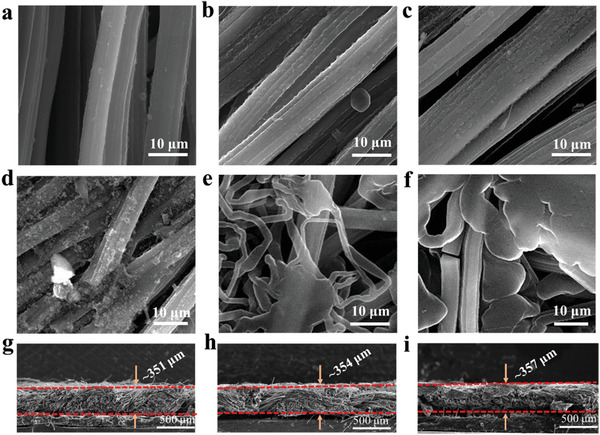
Morphology characterization of the CC@Sb_2_S_3_ and CC with different Li plating capacities. Top‐view SEM images of the CC@Sb_2_S_3_ with a plating capacity of a) 3, b) 5, c) 10 mAh cm^−2^. Top‐view SEM images of the CC with a plating capacity of d) 3, e) 5, f) 10 mAh cm^−2^. Cross‐sectional SEM images of CC@Sb_2_S_3_ with a plating capacity of g) 3, h) 5 and i) 10 mAh cm^−2^.

In order to systematically investigate the Li deposition behavior on CC@Sb_2_S_3_ and bare CC, the Li|CC@Sb_2_S_3_ cells were assembled to test at a galvanostatic discharge with a current density of 1 mA cm^−2^. The voltage–capacity curve of the CC@Sb_2_S_3_ during the Li plating process is shown in Figure S5 (Supporting Information). When the voltage is above 0 V, the reactions that occur include the conversion reaction of Sb_2_S_3_, the alloying reaction as well as the formation of SEI, and when the voltage is reduced below 0 V, Li starts to nucleate and deposit. After Li plating with a capacity of 3 mAh cm^−2^, a dense lithium layer can be detected tightly covered around the carbonaceous core with the guidance of the formed Li–Sb alloy and Li_2_S‐rich SEI, and the thickness of a single fiber increases from 5.4 to 6.5 µm (**Figure** **3**a). In comparison, the bare CC shows loose aggregated lithium chunks due to its lithiophobic nature (Figure 3d) with the same Li plating capacity. As shown in Figure 3b, with increasing the Li deposition capacity to 5 mAh cm^−2^, Li preferentially deposited along the initial dense and even Li layer. The single fiber in the modified CC is tightly wrapped by Li metal with the thickness increasing to 7.7 µm. With further increasing the Li deposition capacity to 10 mAh cm^−2^ (Figure 3c), a large amount of Li can be detected wrapping the single fiber, presenting large lithium masses while no Li dendrite can be observed. At this point, the thickness of the single fiber is ≈10 µm. However, bare CC shows indiscriminate dendritic Li growth at the holes of the fibers when the deposition amount increased to 5 mAh cm^−2^ (Figure 3e), and this phenomenon is much severer with a plating Li capacity of 10 mAh cm^−2^ (Figure 3f). The thickness variation of CC@Sb_2_S_3_ is further detected from the cross‐sectional SEM image with different Li electrodeposition capacities. As displayed, the thickness of the CC@Sb_2_S_3_ (Figure S6, Supporting Information) is ≈341 µm. With different Li deposition capacities, the thickness of CC@Sb_2_S_3_@Li anode is ≈351, 354, and 357 µm, respectively with 3, 5, and 10 mAh cm^−2^ Li deposition (Figure 3g–i), revealing minimal thickness change. The negligible variation in the Li metal thickness indicates that Li metal prefers to spatially confined inside the skeleton rather than stacking on the host surface. Such dense and flat deposition morphology as well as negligible volume change throughout the plating process can be attributed to the synergy of the Sb active sites, Li_2_S‐rich SEI and spatially confinement effect of the 3D architecture contributing to the final homogeneous Li nucleation and growth. Meanwhile, the thickness variation of bare CC with different Li deposition capacity was also be monitored by the SEM technique (Figure S7, Supporting Information). As shown, the bare CC is ≈341 µm. After a Li plating capacity of 3 mAh cm^−2^, the thickness increased to 354 µm. Particularly, with further Li plating capacity to 10 mAh cm^−2^, the thickness of CC increases from 358 (5 mAh cm^−2^) to 412 µm (10 mAh cm^−2^), revealing that the Li metal was growth on the surface on carbon cloth due to the high lithiophobicity of the CC host.

**Figure 4:**
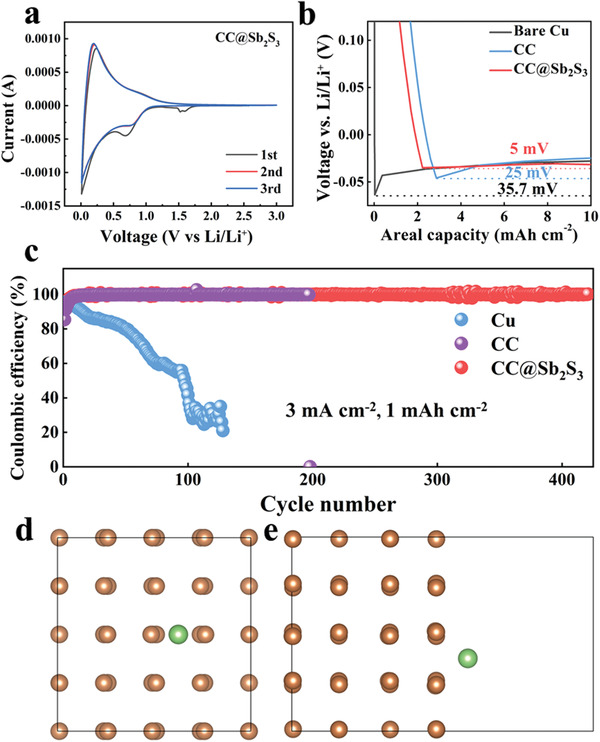
Electrochemical performance and DFT calculation of CC@Sb_2_S_3_. a) CV curves obtained in the potential window range of 0.01–3.00 V at 0.05 mV s^−1^. b) The Li nucleation overpotential comparison. c) CE comparison of CC@Sb_2_S_3_, CC and Cu at 3 mA cm^−2^ with a plating capacity of 1 mAh cm^−2^. The DFT calculation demonstrating the lithiophilicity of Sb. d) The top and e) side view of the most stable site for Li on Sb (100) surface.

The cyclic voltammetry (CV) tests are performed to disclose the role of Sb_2_S_3_ during the lithiation process. **Figure** **4**a shows the CV curves of CC@Sb_2_S_3_. In the cathodic scan, the peak at ≈1.6 V in the first cycle can be attributed to the formation of Li_2_S (Sb_2_S_3_ + 6Li^+^ + 6e^−^→2Sb + 3Li_2_S), and another visible peak at 0.7 V is associated with the generation of Li–Sb alloy by the reaction of 2Sb + 6Li^+^+ 6e^−^→2Li_3_Sb. The peaks located at ≈0.2 V among the cathodic and anodic processes correspond to the intercalation and deintercalation process of Li^+^ and CC. In the anodic process, the peak located at ≈0.9 V is designated to the dealloying process (2Li_3_Sb→2Sb + 6Li^+^+ 6e^−^).^[^ ^38^ ^]^ During the subsequent two cycles, a slight shift of Li–Sb alloy toward higher voltage in the cathodic scan can be detected. High overlapping alloy and dealloy peak profiles demonstrates the reversible alloying reaction during the cycling. With the guidance of Sb active sites and the Li_2_S‐rich SEI, the Li nucleation and growth can be effectively regulated, finally giving a Li dendrite‐free and highly efficient lithium metal battery. Meanwhile, the XRD characterization of the pure Sb_2_S_3_ electrode after three cycles of Li plating/stripping is also conducted. As shown in Figure S8 (Supporting Information), in addition to the three characteristic peaks of the Cu at 43° and the peak of PVDF centered at 20°, the broad peak located at 30° is corresponded to Sb (JCPDS no. 17‐0125), which is highly consistent with the CV results. In addition, the faint Li_2_S diffraction peak at 46° can also be detected.

The Li nucleation behavior on different scaffolds has also been tested. The nucleation overpotential (defined as the potential gap between the initial voltage drop and subsequent smooth voltage plateau) of Li on different substrates is vital for controllable and uniform Li nucleation to achieve high‐performance Li metal battery. As shown in Figure 4b, the nucleation overpotential of CC@Sb_2_S_3_ is only ≈5 mV, much smaller than that on planar Cu (≈36 mV) and bare CC (≈25 mV), which can be attributed to the Sb regulation, the generated Li_2_S‐rich SEI and the reduced local current density. To further verify the role of Sb_2_S_3_ on Li nucleation and growth, CE (defined as the ratio of Li stripping capacity to Li plating capacity in each charging/discharging cycle) can also reflect the reversibility of lithium upon plating and stripping process. The CE test is carried out under different current densities in the ether‐based electrolytes with a fixed capacity of 1 mAh cm^−2^, and then charged to 1 V. At the current density of 1 mA cm^−2^, CE of CC@Sb_2_S_3_@Li still can be maintained at ≈99.7% even after an ultralong cycling over 530 cycles, while the CE of Cu foil degraded rapidly only after 150 cycles (Figure S9, Supporting Information). The initial relative low CE can be ascribed to the consumption of lithium resulted from the solid electrolyte interface formation and the conversion of CC@Sb_2_S_3_ to Li_2_S and Sb. With the current density increased to 3 mA cm^−2^, the CE of CC@Sb_2_S_3_@Li can keep at ≈99.5% as long as 420 cycles in sharp contrast with that of Cu foil faded quickly (Figure 4c). Meanwhile, the CE of bare CC was also tested. As shown, the CE of bare CC can only be stabilized for only ≈197 cycles for the Li dendrite growth induced short‐circuit. The much superior CE of CC@Sb_2_S_3_@Li demonstrates high reversibility of Li plating/stripping on Li anode scaffold, which proves the positive effect of CC@Sb_2_S_3_ and provides strong evidence for uniform deposition of Li and excellent electrochemical performance. Due to the reduction of local current density by the 3D scaffold, the guiding effect of Sb seeds and the efficient ion transport effect of Li_2_S‐rich SEI, the nucleation and growth of lithium is uniform. These favorable results further demonstrate that CC@Sb_2_S_3_@Li can serve as a feasible scaffold for lithium to construct high‐performance Li metal batteries. Density functional theory (DFT) calculations were also conducted to further demonstrate the improved lithiophilicity with the preplanted of Sb sites (Figure 4d,e). As shown, Li has a much lower binding energy value (−1.74 eV) to the Sb (100) surface compared to Li and graphene (−1.38 eV), revealing a much improved lithiophilicity.^[^ ^25^ ^]^ As a result, preplanted Sb sites can significantly improve the lithiophilicity of CC and guarantee the stable cycling of the hosted Li metal anode. Meanwhile, the experimental test of the Li wettability of CC@Sb_2_S_3_ compared with bare CC was also carried out. As shown in Figure S10 (Supporting Information), molten Li can quickly climb up the host and be fully immersed with metallic Li quickly within 17 s owing to the superior lithiophilicity of the CC@Sb_2_S_3_ matrix. As a sharp contrast, molten Li could not wet the bare CC even after several minutes. The results above can firmly testify to the superior lithiophilicity of CC@Sb_2_S_3_.

**Figure 5:**
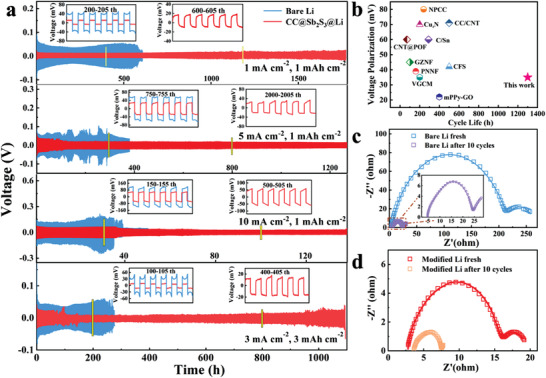
Electrochemical performance of the symmetric cells. a) Galvanostatic cycling performance of Li symmetric cells at different current densities and capacities. b) Electrochemical performance comparison with the reported works at 5 mA cm^−2^ with a fixed capacity of 1 mAh cm^−2^. The electrochemical impendence spectra of symmetric cells before and after cycling with c) bare Li and d) CC@Sb_2_S_3_@Li.

The galvanostatic cycling performance of CC@Sb_2_S_3_@Li is investigated in symmetric cells at gradient current densities and the results are shown in **Figure** **5**. As shown in Figure 5a, the cell with CC@Sb_2_S_3_@Li anode exhibits an ultralong stable cycling over 1750 h with a quite low overpotential (≈12 mV). In contrast, the voltage polarization of cell with plain Li foil can only be stabled at 40 mV for the first 120 cycles and a gradual increase in voltage polarization afterward can be clearly detected with a lifetime of only 600 h, revealing the formation of excessively thick SEI and the severe Li dendrite growth. As the current density increases to 3 mA cm^−2^, CC@Sb_2_S_3_@Li electrode still shows a low overpotential and an ultra‐long lifespan over 1200 h in sharp contrast to that with plain Li foil (Figure S11, Supporting Information). Impressively, cell with CC@Sb_2_S_3_@Li displays surprisingly cycle stability over 700 cycles, with low overpotential of ≈50 mV even at a high current density of 10 mA cm^−2^. While the counterpart with Li foil shows significant voltage fluctuations with limited cycling life owing to the growth of Li dendrite and pulverization of electrode. Moreover, the symmetric cell with CC@Sb_2_S_3_@Li anode at 3 mA cm^−2^ with a high stripping/plating capacity of 3 mAh cm^−2^ could maintain excellent stability for more than 1000 h. In addition, the electrochemical rate performance of Li symmetric batteries was also tested, and the results are shown in Figure S12 (Supporting Information). When the current density increases from 0.5 to 10 mA cm^−2^, the symmetric cell with CC@Sb_2_S_3_@Li electrode displays steady and much lower overpotential. While, the symmetric battery with plain Li foil exhibits a large voltage fluctuation, resulting from serious growth of Li dendrite. Figure 5b summarizes the electrochemical performance comparison of CC@Sb_2_S_3_@Li anode with the reported literature at 5 mA cm^−2^ applying the ether‐based electrolyte.^[^ ^39^, ^40^, ^41^, ^42^, ^43^, ^44^, ^45^, ^46^, ^47^ ^]^ As exhibited, the performance of CC@Sb_2_S_3_@Li far exceeds the published works in terms of its ultralong cycle life (≈3200 cycles) and small polarization voltage (≈18 mV). In addition, the results achieved in the recently reported literature on the construction of 3D structured Li anode are cited (Table S1, Supporting Information), and the comparative results show that the method used in this study demonstrates excellent advantages in terms of both cycle life and polarization voltage. To prove the feasibility of CC@Sb_2_S_3_@Li in carbonate electrolyte, the symmetric cells with carbonate electrolyte were also assembled and tested at 3 mA cm^−2^, 1 mAh cm^−2^. As shown in Figure S13 (Supporting Information), cells with CC@Sb_2_S_3_@Li show a much lower polarization voltage of ≈60 mV. As a contrast, cells with bare Li foil demonstrate a much higher polarization voltage of ≈91 mV and fast polarization increase can be detected only after ≈20 cycles. In order to pursue high specific energy density battery, the CC@Sb_2_S_3_ host with a thickness of 240 µm is also prepared by the rolling treatment (Figure S14, Supporting Information). The electrochemical performance of the CC@Sb_2_S_3_@Li anode with the rolled host was also tested in carbonate electrolyte. As Figure S15 (Supporting Information) exhibited, the cell with CC@Sb_2_S_3_@Li still shows much lower voltage hysteresis and much longer cycling life than that with bare Li foil. Restricted by the high thickness and density of carbon materials applied in our work, there exists a certain degree of sacrifice of the specific capacity of Li metal anode. However, the obtained composite Li anode shows superior electrochemical performance and particularly superior safety than the plain Li foil. To unveil the reason for the long‐term stable cycling performance of the Li symmetric cell, the electrochemical impedance spectroscopy (EIS) of the symmetric cells before and after cycling are also tested. As shown in Figure 5c,d, the semicircle at the high‐frequency region of the Nyquist curve can be designated to the interfacial resistance (*R*_SEI_) between the lithium metal and the SEI layer, and its value is closely related to the transport kinetics of lithium ions. The EIS simulation results are shown in Table S2 (Supporting Information) with the simulation circuit (Figure S16, Supporting Information). For the fresh symmetric cell with plain Li foil, the *R*_SEI_ is ≈212.7 Ω, which is ascribed to the original oxide layer of Li, and the *R*_SEI_ reduces to ≈3.6 Ω owing to enlarged area of the Li dendrite and volume change after 10 cycles. However, the cells with CC@Sb_2_S_3_@Li show a lower value of *R*_SEI_ (≈13.5 Ω) and reduce to ≈2.7 Ω after 10 cycles, resulting from the in situ produced high ionic conductive Li_2_S‐rich SEI. The enhanced interfacial stability and ionic conductivity of Li_2_S‐rich SEI can facilitate the fast Li plating/stripping kinetics, which is mutually confirmed by the voltage polarization of modified symmetric cells over prolonged cycles. To prove the dendritic Li suppression on CC@Sb_2_S_3_ scaffold, SEM was used to characterize the morphology of electrodes after cycling. After 10 cycles at 1 mA cm^−2^, 1 mAh cm^−2^, the surface of plain Li foil presents high surface area due to rough dendritic Li growth, which will not only accelerate the consumption of electrolyte but also lead to the pulverization and rapid failure of electrode (Figure S17a, Supporting Information). In contrast, CC@Sb_2_S_3_@Li electrode demonstrates smooth and compact Li deposition (Figure S17b, Supporting Information), revealing a promising practical application prospect. The homogeneous Li deposition can be attributed to the homogeneous Li^+^ flux guided by the nano Sb active sites and Li_2_S‐rich SEI.

**Figure 6:**
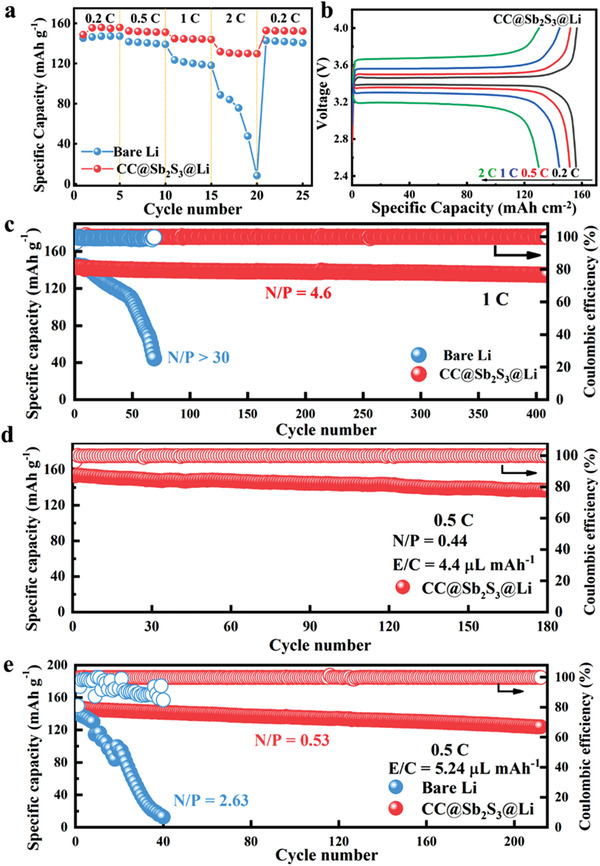
The electrochemical performance of cells coupled with different cathodes. a) Rate performance of Li|LFP. b) Voltage profiles of CC@Sb_2_S_3_@Li|LFP. c) Long‐term cycling performance of CC@Sb_2_S_3_@Li|LFP and Li|LFP full cells at 1 C. The cycling performance of full cells under more realistic conditions coupled with d) LFP and e) NCM523 cathode under lean electrolyte and low N/P at 0.5 C.

To further testify the practical applications, full cells were assembled with commercially available LFP cathodes (≈13.36 mg cm^−2^). CC@Sb_2_S_3_@Li was achieved by prelithiation with a capacity of 10 mAh cm^−2^. **Figure** **6**a shows the rate performance of the full cells. It can be clearly detected that cells with CC@Sb_2_S_3_@Li show superior rate performance compared with those with the plain Li foil. At 0.2 C (1 C = 170 mAh g^−1^), CC@Sb_2_S_3_@Li|LFP exhibits a high discharge capacity of 155 mAh g^−1^. When the discharge rate increased to 1 and 2 C, high capacities of 148 and 125 mAh g^−1^ still can be yielded, and the polarization voltage exhibits an acceptable range of increase due to the rapid increase of the current density with high loading LFP cathode (Figure 6b). As a contrast, cells with plain Li foil exhibit a lower discharge capacity, as evidenced by the more pronounced voltage polarization especially at high discharge rates of 1 C and 2 C (Figure S18, Supporting Information). And a sharp decay in the capacity can also be detected at 2 C. Meanwhile, the long‐term cycling performance of Li|LFP at 1 C was also investigated. The superior cycling performance (over 410 cycles) and a high capacity retention up to 94% under a low N/P (defined as the ratio of negative to positive capacity) of ≈4.6 can be achieved in CC@Sb_2_S_3_@Li|LFP cell (Figure 6c). In contrast, the Li|LFP cell with excess Li (N/P>30) and flooded electrolyte shows a sharp capacity degradation only after very finite 20 cycles, mainly due to the growth of Li dendrite, poor electrode/electrolyte interface stability, and fast electrolyte consumption. As known, the electrochemical performance of Li metal anode is usually tested with largely excess Li metal (over 10‐fold) and much flooded electrolyte, which not only is a waste of resources, but also add hazardous weight and significantly compromise the overall specific energy of cell. In order to pursue high‐energy‐density batteries to meet the demand of large‐scale energy storage while improving the lithium utilization, the amount of lithium metal and electrolyte should be strictly restricted (i.e., N/P ≤ 2, E/C defined as the ratio of electrolyte to capacity ≤ 5 µL mAh^−1^). Batteries with low N/P and E/C ratios inevitably impose much higher requirements on Li metal anode considering the inherent side reactions of metallic Li with the liquid electrolyte. To unveil the practical application of the modified Li metal, CC@Sb_2_S_3_@Li is further evaluated under a more realistic condition of lean electrolyte and limited Li metal. When the capacity of anode and the amount of electrolyte used are controlled at a lower level (N/P: ≈ 1.32 and E/C: ≈4.4 µL mAh^−1^), the Li|LFP with modified electrode maintains a steady‐going charge–discharge cycles up to 350 cycles with a capacity retention as high as ≈86.5% at 0.5 C (Figure S20, Supporting Information). In contrast, the Li|LFP cell with plain Li foil can only survive for only 20 cycles although with a higher Li content (N/P: ≈2.2), resulting mainly from the fast consumption of electrolyte and metallic Li. Furthermore, CC@Sb_2_S_3_@Li|LFP still delivers a high specific capacity of 152.8 mAh g^−1^ at the first cycle under more harsh conditions (the N/P: ≈0.44, E/C: ≈4.38 µL mAh^−1^) (Figure 6d). And an excellent cycling over 180 cycles with a remarkable capacity retention as high as 90% at 0.5 C can be maintained. The excellent cycling performance of CC@Sb_2_S_3_@Li under harsh condition can be attributed to the synergy of Li_2_S‐rich SEI layer and lithophilic Sb seeds, guaranteeing the highly reversible Li plating/stripping and the uniform Li nucleation/growth. Also, the 3D conductive scaffold not only facilitates to alleviate the volume expansion but also reduces the local current density. All the merits above finally afford the excellent performance Li metal anode. Moreover, the generalizability of CC@Sb_2_S_3_@Li is also evaluated via coupling with high‐voltage and high‐loading NCM523 cathode (≈1.98 mAh cm^−2^). As shown in Figure 6e, the CC@Sb_2_S_3_@Li|NMC523 cell provides a high specific capacity of 146.2 mAh g^−1^ at the initial cycle, and a superior capacity retention of ≈85% can be achieved after 212 cycles under a more realistic condition (N/P: ≈0.53 and E/C: ≈5.24 µL mAh^−1^) at 0.5 C (1 C = 155 mAh g^−1^). In contrast, the discharge capacity of the Li|NMC523 cell decays fast. To disclose the reason for the excellent cycling performance of CC@Sb_2_S_3_@Li, XPS and EIS analysis of the cycled Li anodes were carried out. As for cell with plain Li foil, the interfacial resistance of cells reduced from ≈435 (the fresh cell) to ≈265 Ω after 10 cycles, and then sequentially reduced to ≈160 Ω (50^th^ cycle) ascribed to the Li dendrite growth with increased active surface area (Figure S21a, Supporting Information). As a contrast, the fresh cell with CC@Sb_2_S_3_@Li demonstrates much lower interfacial resistance compared with that with plain Li foil. And much smaller interfacial resistance still be kept after 50 cycles. The significantly reduced interfacial resistance can be attributed to the fast charge transfer with the unique host (Figure S21b, Supporting Information). Also, cell with CC@Sb_2_S_3_@Li shows stable and small interfacial resistance over Li plating/stripping cycles, revealing the much more stable electrolyte/electrode interface with the CC@Sb_2_S_3_@Li anode. The stable electrolyte/electrode interface contributes to the low cell polarization and the subsequently superior cell cycling performance. Meanwhile, the XPS characterization of cycled Li anode was also carried out and the results were shown in Figure S22 (Supporting Information). The C 1s spectra show three main peaks at 290.2, 288.7, 286.8, and 284.8 eV corresponding to signals of CO_3_^2−^, COOR, C—O, and C—C, respectively. The cell with CC@Sb_2_S_3_@Li shows much lower signals compared with that with plain Li foil, indicating the much serious reaction of electrolyte with plain Li foil. The fast consumption of electrolyte and metallic Li finally results in the fast cell decay. The rational design of Li anode can stimulate its practical application toward safe, stable, and long lifespan Li metal batteries. Notably, the performance of CC@Sb_2_S_3_@Li under harsh conditions far exceeds the majority of recently published works,^[^ ^48^, ^49^, ^50^ ^]^ demonstrating a promising prospect for practical usage.

## 3 Conclusion

In summary, multifunctional Sb_2_S_3_ decorated CC is facilely synthesized by a simple one‐step hydrothermal synthesis, which is rationally exploited as the scaffold to stabilize metallic Li anode. Li_2_S‐rich artificial solid electrolyte interface layer combined with the lithiophilic Sb active sites can be in situ generated, which can guarantee the highly reversible Li plating/stripping and the homogeneous Li nucleation/growth. Consequently, the symmetric cell assembled with CC@Sb_2_S_3_@Li achieves an ultralong cycling lifespan (over 3200 cycles) of Li plating/stripping with a very low polarization overpotential (≈18 mV) at 5 mA cm^−2^. More importantly, the achieved full cells coupled with LFP/NCM523 both demonstrate outstanding cycling stability even under the more realistic condition of very low N/P with lean electrolyte. This simple host design reveals the feasibility of scale‐up Li metal anode toward practical usage.

## 4 Experimental Section

### Synthesis of CC@Sb_2_S_3_

The CC@Sb_2_S_3_ composites were prepared by a facile, one‐pot hydrothermal method. Before the synthesis operation, acetone, ethanol, and deionized water should be used to purify the CC for three times to remove the surface impurity. In a typical synthesis, solution A: SbCl_3_ (0.2 g) was dispersed in 25 mL deionized water. Then, thiourea (CH_4_N_2_S) (0.17 g) was dispersed in 25 mL deionized water, denoted as solution B. After 2 h stirring, solution A was quickly added to solution B obtaining a uniform solution, which was then transferred to a Teflon‐lined stainless‐steel autoclave (100 mL in volume) preloaded with cleaned CC and heated at 180 °C for 12 h. After cooling to room temperature, the collected modified CC was washed several times with alcohol and deionized water, and then dried at 60 °C for 12 h, denoting as CC@Sb_2_S_3_. Pure Sb_2_S_3_ is synthesized by the same preparation method.

### Fabrication of Electrodes

CC@Sb_2_S_3_@Li was fabricated by assembling CR‐2032 type coin cells, in which CC@Sb_2_S_3_ as the working electrode and Li foil as the reference electrode with Celgard 2325 as the separator. For exploring the electrodeposition process of CC@Sb_2_S_3_ electrode, the morphology change of surface morphology with different lithium deposition capacity was investigated at the condition of current density of 1 mA cm^−2^. Sb_2_S_3_ electrode was prepared by blending the obtained Sb_2_S_3_ powder, Super P, and polyvinylidene fluoride (PVDF) binder with the weight ratio of 8:1:1 in N‐methyl‐2‐pyrrolidone (NMP) solvent. For standardization, 50 µL of 1 m LiTFSI in 1,3‐dioxolane (DOL) and dimethoxy ethane (DME) (1:1 by volume) with 1 wt% LiNO_3_ was used as electrolyte for both CE and symmetric cell tests. CV and EIS data were obtained on CHI660e electrochemical workstation and Gamry respectively.

### Characterizations

The X‐ray diffraction analyzer (XRD, bruker d8‐Advanced) was used for the physical phase identification with a mono chromated Cu K*α* radiation. A field emission scanning electron microscope (SEM, INCA X‐Max 80) as well as a field emission transmission electron microscope (TEM, JEM‐2100F) were used to characterize the samples for morphology. X‐ray photoelectron spectroscopy (XPS) analysis of the electrodes was performed using a Thermo ESCALAB 250XI model X‐ray photoelectron spectrometer, while the C1s peak at 284.8 eV was used as a reference for calibration. For postcycle electrodes, surface cleaning using DME is required after disassembly of the cell, dried, and transferred for further characterization.

### Full Cell Assembly and Electrochemical Measurement

The commercial LFP (≈13.36 mg cm^−2^) and NCM523 (≈12.25 mg cm^−2^) electrodes with ultrahigh active matter mass loading were supplied by MTI. The Li|LFP and Li|NCM523 cells (CR‐2032 type) were assembled in an argon‐filled glove box with Celgard 2500 as a separator, using electrolytes of 1 m LiPF_6_ in EC/EMC and 1 m LiPF_6_ in EC/DEC/EMC, respectively.

When CC@Sb_2_S_3_@Li anode with an areal capacity of 10 mAh cm^−2^ was paired with LFP cathode with a surface capacity of 2.27 mAh cm^−2^ under conventional test conditions (N/P: ≈4.4), the volume of electrolyte was 60 µL. In contrast, the same volume of electrolyte was used to prepare Li|LFP full cells with lithium foils as the anode, but the N/P was much higher than 30. The more severe test conditions were achieved by reducing the capacity of the anode Li and the amount of electrolyte used, with the capacity of the anode Li reduced to 3 and 1 mAh cm^−2^, respectively, and the amount of electrolyte added as low as 5 µL. The charge–discharge cycle performance of Li|LFP battery was tested in the voltage range of 2.5–4 V. The charge/discharge tests of Li|NCM523 cell was tested at the voltage range of 3–4.2 V. The conductivity studied using AC impedance spectroscopy was recorded in the frequency range of 0.10 Hz–7 MHz by Biologic VSP potentiostat. The electrochemical properties of all the cells were tested by LAND and NEWARE cell test systems.

### Simulation

The theoretical calculations were conducted using the DFT method via Vienna ab initio simulation package (VASP) code.^[^ ^51^ ^]^ The electron–ion interactions were depicted by the projector‐augmented wave (PAW) method.^[^ ^52^ ^]^ The generalized gradient approximation (GGA) of Perdew‐Burke‐Ernzerhof (PBE)^[^ ^53^ ^]^ was utilized to describe the exchange‐correlation functional. The cutoff energy for plane‐wave basis and k‐point mesh for surface structure were tested to be 400 eV and 5 × 5 × 1, respectively. For all calculations, the force convergence criterion was 0.01 eV Å^‐1^, and the energy convergence criterion was 10^−6^ eV. The binding energy of Li to Sb (100) is defined by

(1) $$E_{b}=E_{\mathrm{surface}+\mathrm{Li}}-E_{\mathrm{surface}}-E_{\mathrm{Li}}$$

where *E*_surface + Li_ and *E*_surface_ refer to the energy of Sb (100) absorbing a Li atom and the energy of Sb (100) respectively, and *E*_Li_ is the energy of a Li atom in Li metal.

## Conflict of Interest

The authors declare no conflict of interest.

## Supporting information

Supporting Information

## Data Availability

Research data are not shared.
