# Supplementary material for: In Situ Construction of Efficient Interface Layer with Lithiophilic Nanoseeds toward Dendrite‐Free and Low N/P Ratio Li Metal Batteries
Source: Adv Sci (Weinh). 2022 Jan 25;9(8):2104391. doi: 10.1002/advs.202104391 (PMC8922099; doi:10.1002/advs.202104391)
Supplement: Supplementary file 1 — Supporting Information [file ADVS-9-2104391-s001.pdf]

ADVANCED SCIENCE
Open Access

Supporting Information

for *Adv. Sci.*, DOI 10.1002/advs.202104391

In Situ Construction of Efficient Interface Layer with Lithiophilic Nanoseeds toward Dendrite-Free and Low N/P Ratio Li Metal Batteries

Lingli Luo, Shuixin Xia, Xun Zhang, Junhe Yang and Shiyou Zheng**

ADVANCED SCIENCE
Open Access

Supporting Information

for *Adv. Sci.,* DOI: 10.1002/advs.202104391

In-situ construction of efficient interface layer with lithiophilic nanoseeds toward dendrite-free and low N/P ratio Li metal batteries

Lingli Luo, Shuixin Xia, Xun Zhang, Junhe Yang, Shiyou Zheng**

Supplementary Materials for

In-situ construction of efficient interface layer with lithiophilic nanoseeds toward dendrite-free and low N/P ratio Li metal batteries

Lingli Luo, Shuixin Xia*, Xun Zhang, Junhe Yang, Shiyou Zheng*

Contents

1. Supporting figures

Figure S1. The XPS survey of CC@Sb_2S_3.
Figure S2. The EDS of the CC@Sb_2S_3 of SEM.
Figure S3. a) The SEM image of bare CC. b) the diameter distribution of carbon fiber.
Figure S4. The XRD pattern of CC@Sb_2S_3@Li and bare CC.
Figure S5. The illustration of the discharge/charge process of the CC@Sb_2S_3 electrode during the Li plating process.
Figure S6. The cross-sectional SEM image of bare CC@Sb_2S_3.
Figure S7. Cross-sectional SEM images of a) bare carbon cloth and bare carbon cloth with a Li deposition capacity of b) 3, c) 5 and d) 10 mAh cm^{-2}.
Figure S8. The XRD pattern of the bare Sb_2S_3 of Li|Sb_2S_3 cell after 3 cycles at 2mA cm^{-2} and 2 mAh cm^{-2}.
Figure S9. CE comparison of CC@Sb_2S_3 and Cu foil at 1 mA cm^{-2} with a capacity of 1 mAh cm^{-2} and in the ether electrolyte.
Figure S10. The digital photographs of Li infusion process. a) CC@Sb_2S_3; b) bare CC.
Figure S11. Galvanostatic cycling performance of Li symmetric cells at current densities of 3 mA $cm^{-2}$with a fixed capacity of 1 mAh cm^{-2}.
Figure S12. Rate performance comparison of these two symmetric cells at various current densities from 0.5 to 10 mA cm^{-2}.
Figure S13. Voltage profiles of symmetric cells with bare Li and CC@Sb_2S_3@Li at 3 mA cm^{-2} and 1 mAh cm^{-2} in the carbonate electrolyte.
Figure S14. Optical photos of CC@Sb_2S_3 a) before rolling and b) after rolling.
Figure S15. Voltage profiles of symmetric cells with bare Li and CC@Sb_2S_3@Li at 1 mA cm^{-2}, 1 mAh cm^{-2} in the carbonate electrolyte.
Figure S16. EIS equivalent circuit simulation diagram.
Figure S17. The SEM images of (a) bare Li and (b) the CC@Sb_2S_3@Li electrode after 10 cycles at 1 mA cm^{-2} and 1 mAh cm^{-2}.
Figure S18. Voltage profiles of Li|LFP full cell at different C rates.

Figure S19. Long-term cycling performance of Li|LFP cells with plain Li foil at 1 C.
Figure S20. Long-term cycling performance of CC@Sb_2S_3@Li|LFP and Li|LFP full cells at 0.5 C under lean electrolyte and low N/P.
Figure S21. The cycling performance of full cells under more realistic conditions coupled with NCM523 cathode under lean electrolyte (4.4 µl mAh^{-1}) and low N/P (~0.44) at 0.5 C. The electrochemical impendence spectra of a) Li|NCM523 and b) CC@Sb2S3@Li|NCM523 cells after different cycles.
Figure S22. The XPS analysis of C 1s of different Li anodes in Li|NCM523 full cell after 50 cycles at 0.5 C.

2. Supporting tables

Table S1. Comparison of electrochemical performances of symmetric cells with different 3D Li host.
Table S2. Impedance fitting results for different electrodes before and after cycling.
Table S3. Comparison of electrochemical performances of Li|LFP cells.

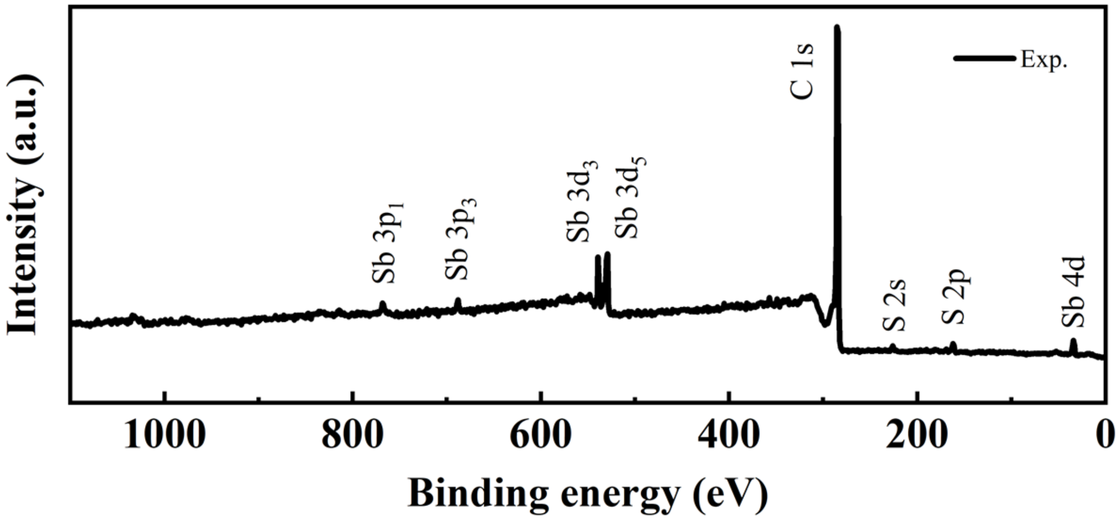

Figure S1. The XPS survey of CC@Sb_2S_3.

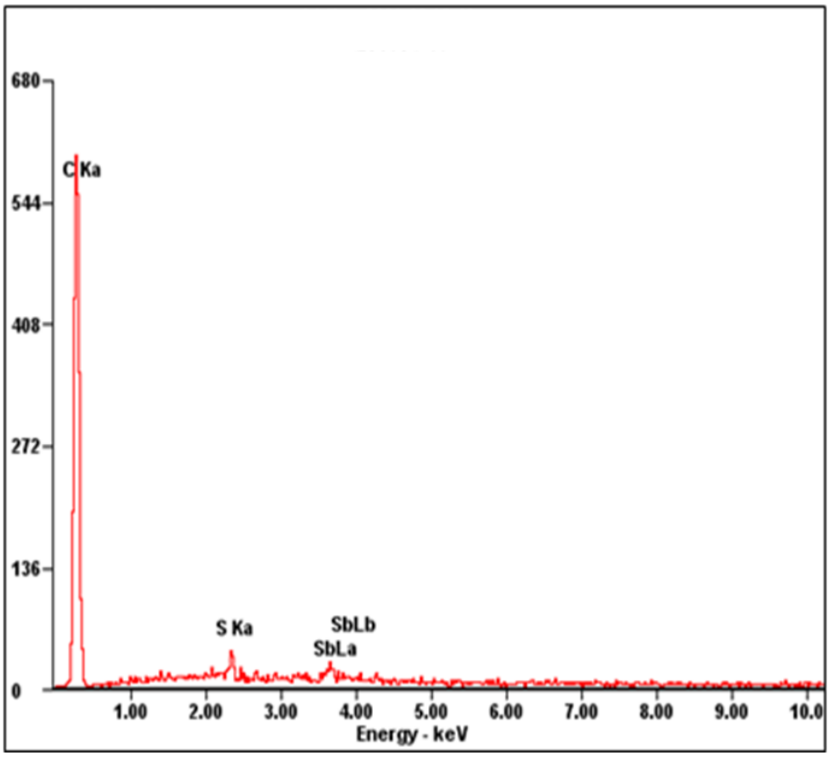

Figure S2. The EDS of the CC@Sb_2S_3 of SEM.

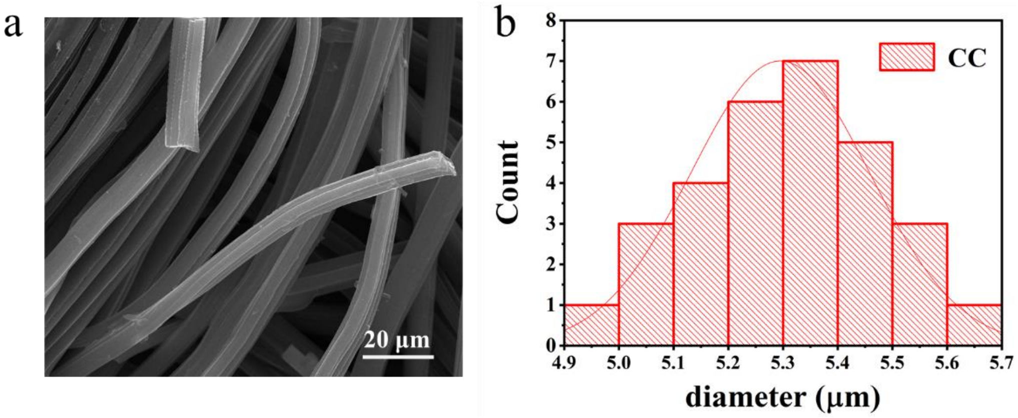

Figure S3. a) The SEM image of bare CC, b) the diameter distribution of carbon fiber.

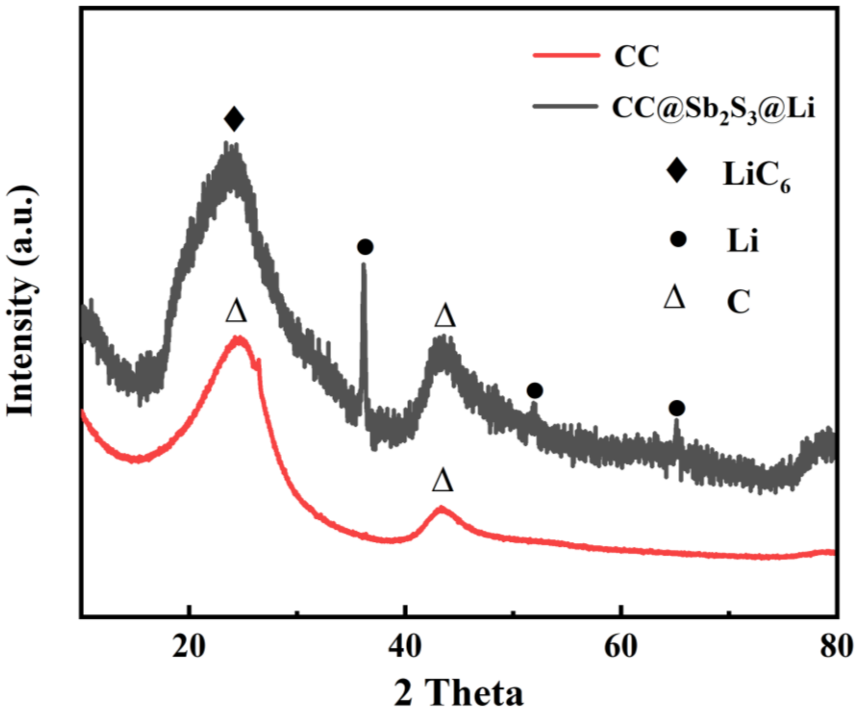

Figure S4. The XRD pattern of CC@Sb_2S_3@Li and bare CC.

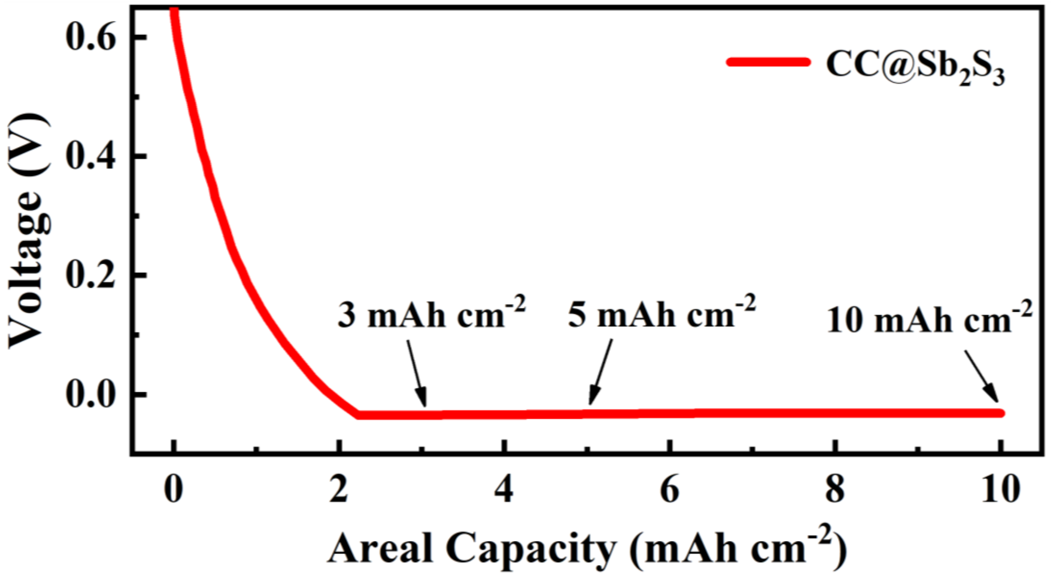

Figure S5. Voltage-capacity curve of the CC@Sb_2S_3 electrode during the Li plating process.

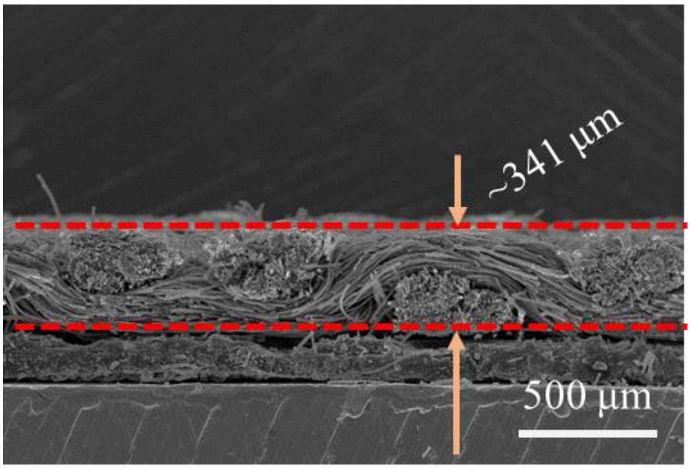

Figure S6. The cross-sectional SEM image of $CC@Sb_2S_3$.

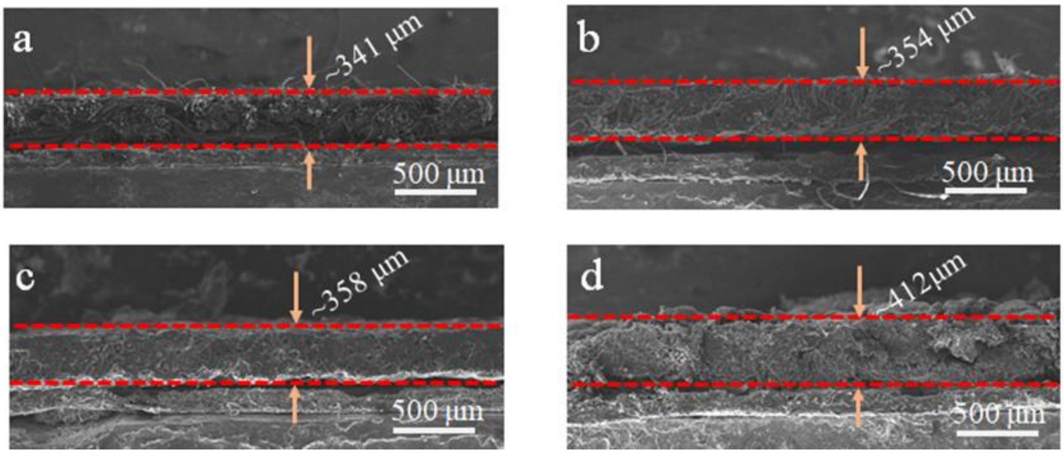

Figure S7. Cross-sectional SEM images of a) bare carbon cloth and bare carbon cloth with a Li deposition capacity of b) 3, c) 5 and d) 10 mAh cm^{-2}.

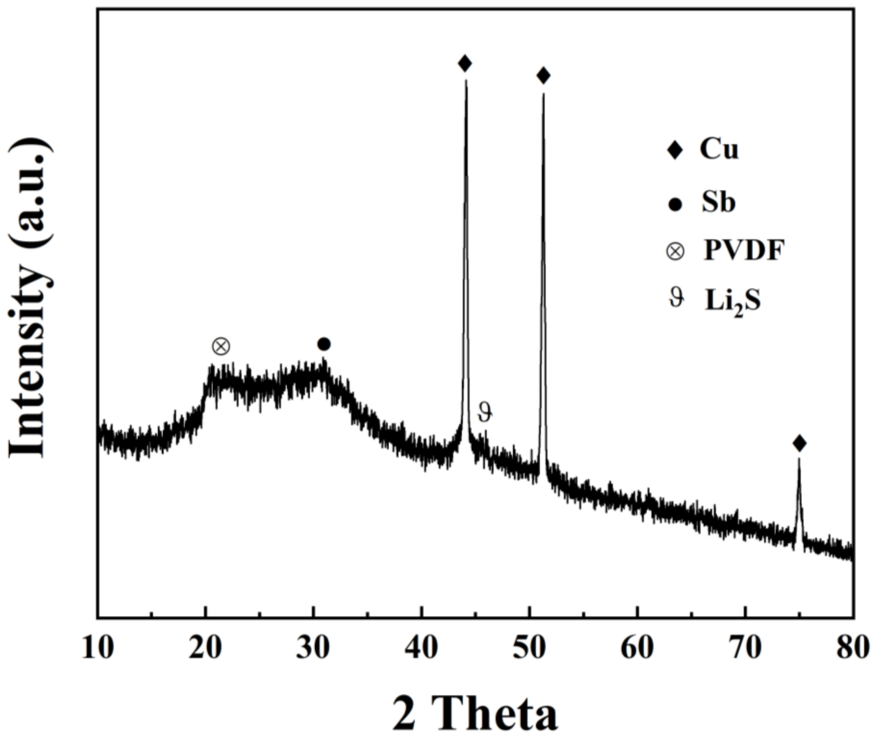

Figure S8. The XRD pattern of the bare Sb_2S_3 electrode of Li|Sb_2S_3 cell after 3 cycles at 2 mA cm^{-2} and 2 mAh cm^{-2}.

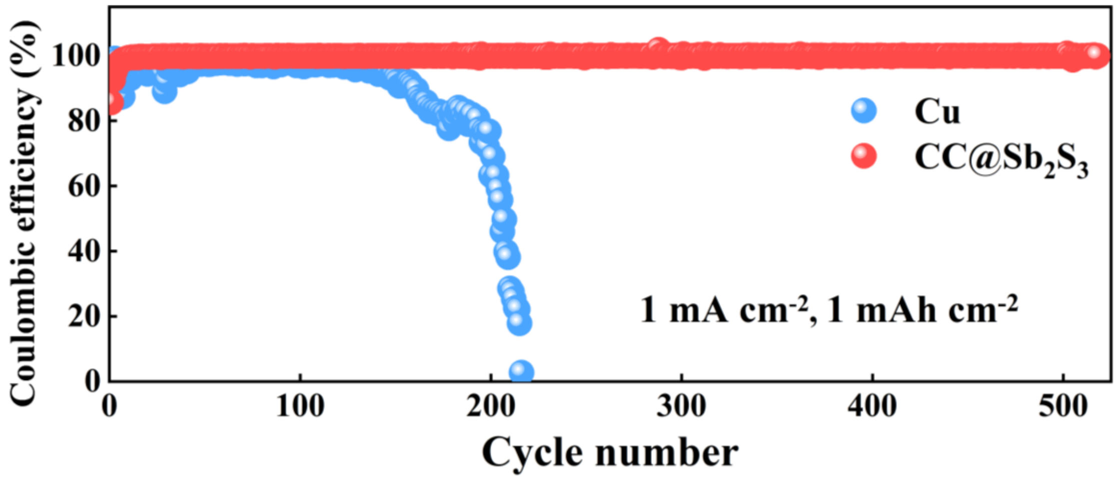

Figure S9. CE comparison of CC@Sb_2S_3 and Cu foil at 1 mA cm^{-2} with a capacity of 1 mAh cm^{-2} and in the ether electrolyte.

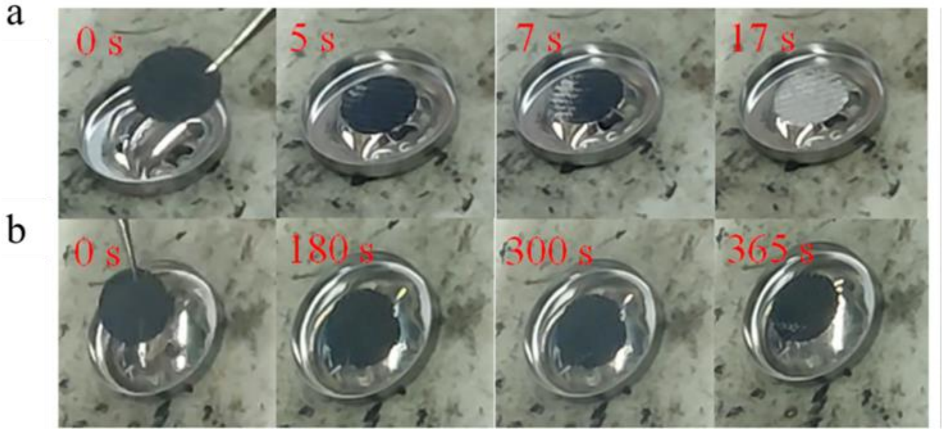

Figure S10. The digital photographs of Li infusion process. a) CC@Sb_2S_3; b) bare CC.

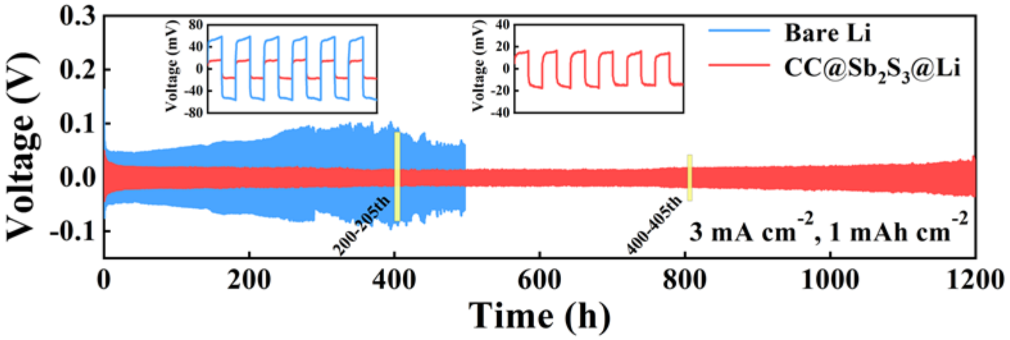

Figure S11. Galvanostatic cycling performance of Li symmetric cells at current densities of 3 mA cm^{-2} with a fixed capacity of 1 mAh cm^{-2}.

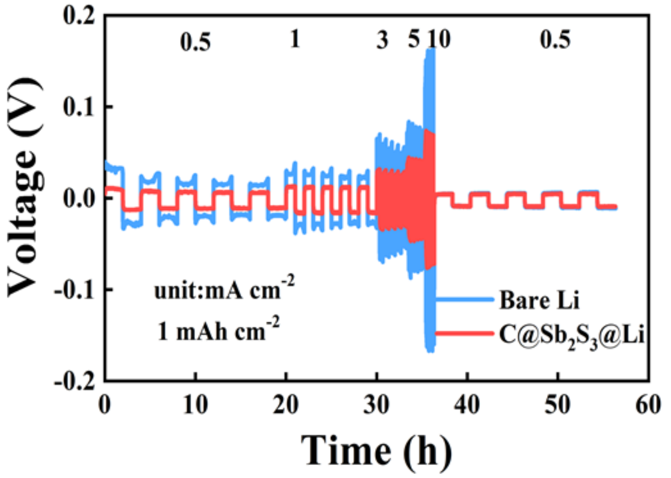

Figure S12. Rate performance comparison of cells at various current densities from 0.5 to 10 mA cm^{-2}.

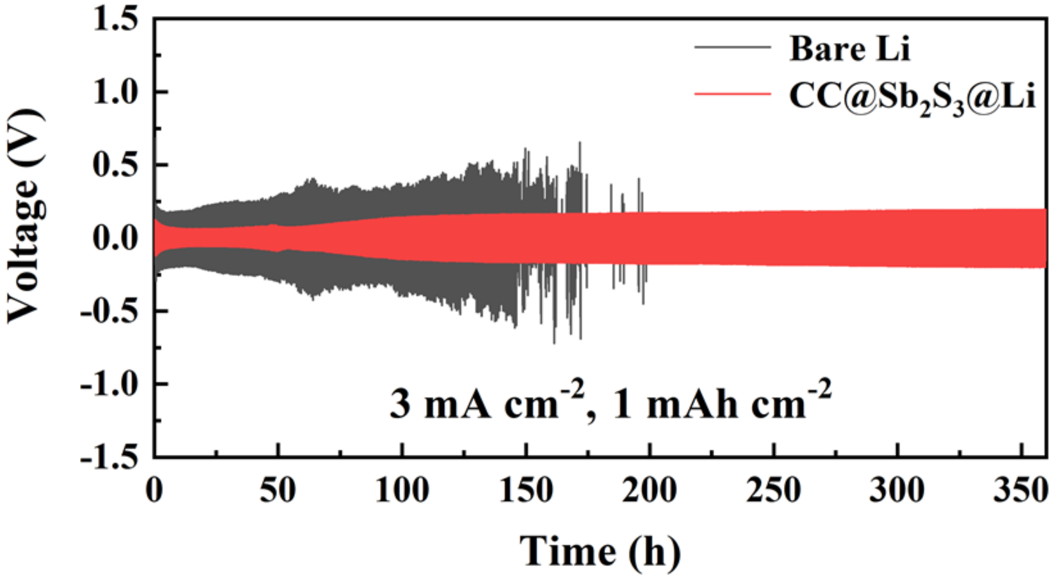

Figure S13. Voltage profiles of symmetric cells with bare Li and CC@Sb_2S_3@Li at 3 mA cm^{-2} and 1 mAh cm^{-2} in the carbonate electrolyte.

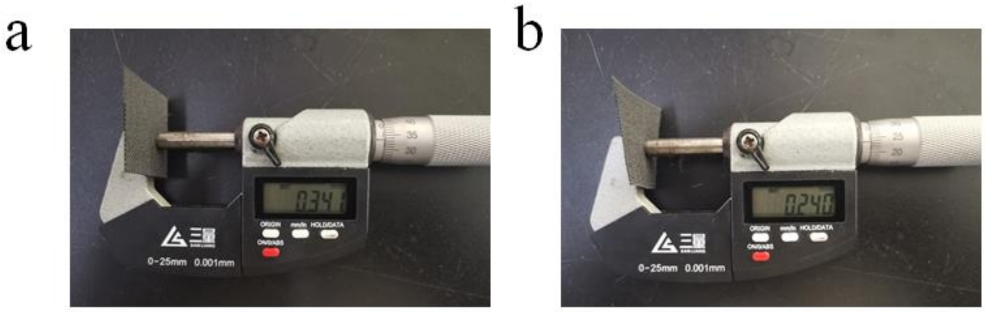

Figure S14. Optical photos of CC@Sb_2S_3 a) before rolling and b) after rolling.

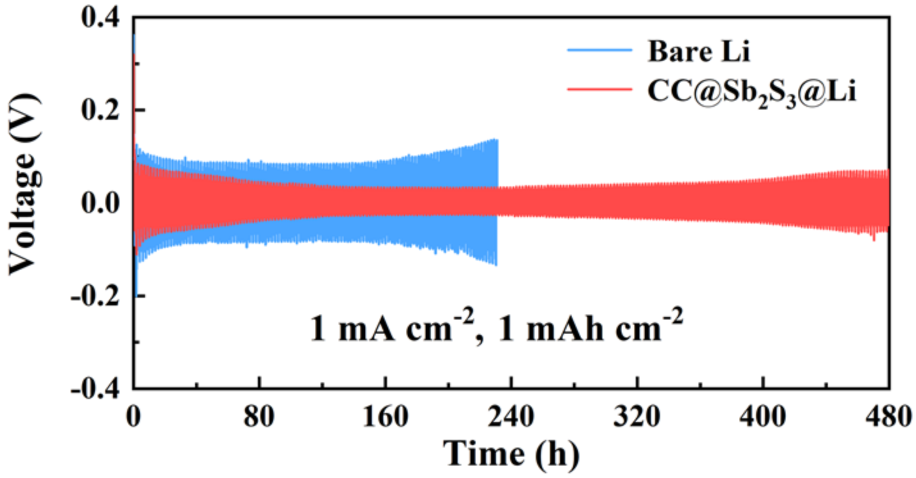

Figure S15. Voltage profiles of symmetric cells with bare Li and CC@Sb_2S_3@Li at 1 mA cm^{-2}, 1 mAh cm^{-2} in the carbonate electrolyte.

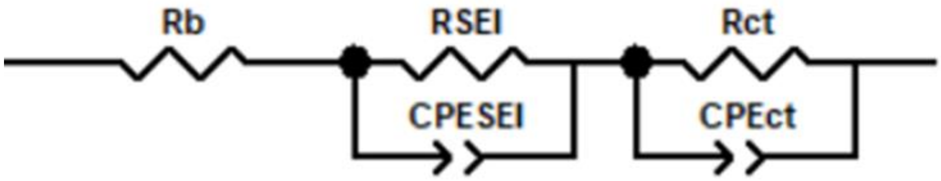

Figure S16. EIS equivalent circuit simulation diagram.

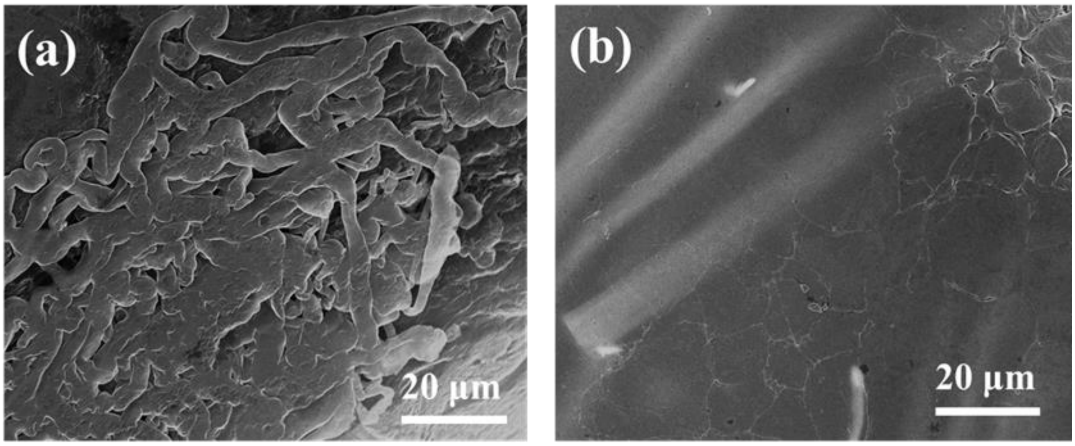

Figure S17. The SEM images of (a) bare Li and (b) the CC@Sb_2S_3@Li electrode after 10 cycles at 1 mA cm^{-2} and 1 mAh cm^{-2}.

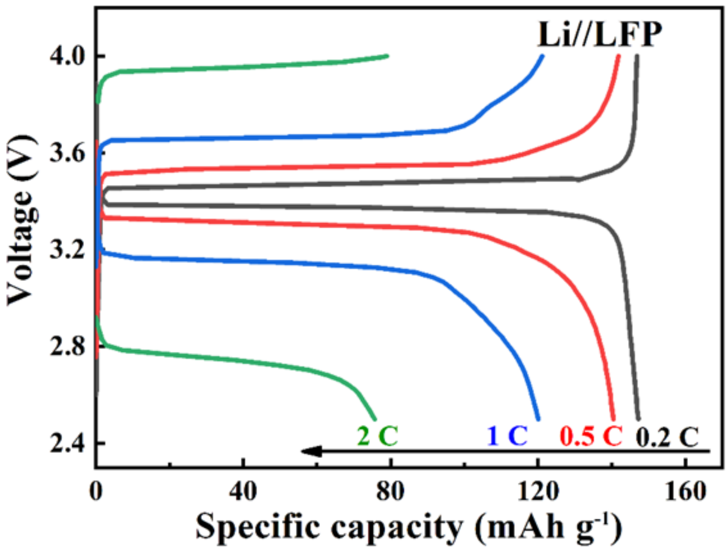

Figure S18. Voltage profiles of Li|LFP full cell at different C rates.

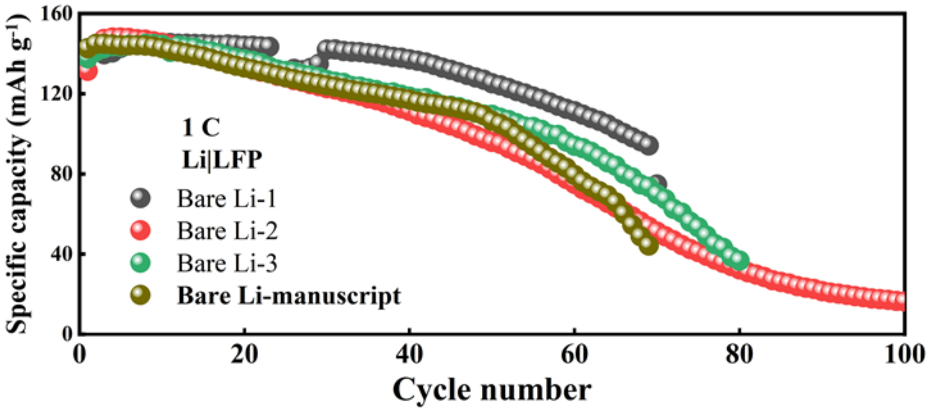

Figure S19. Long-term cycling performance of Li|LFP cells with plain Li foil at 1 C.

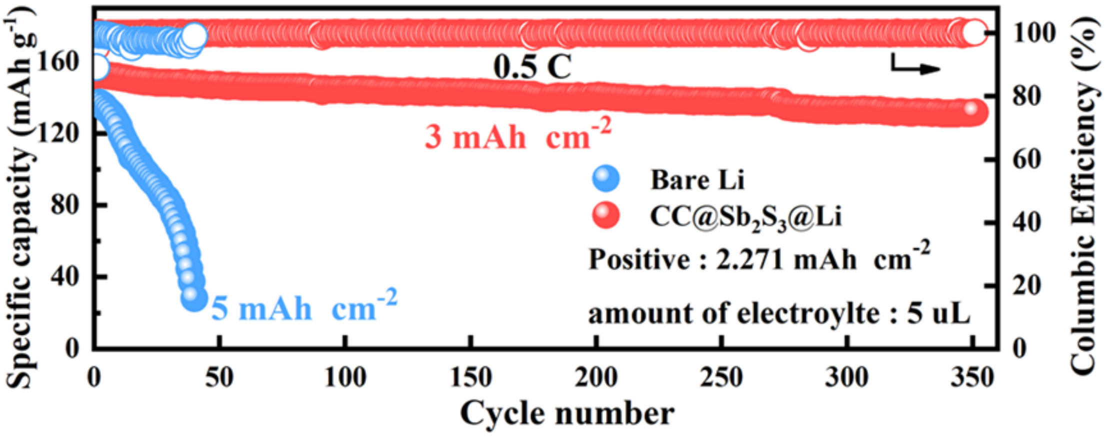

Figure S20. Long-term cycling performance of CC@Sb_2S_3@Li|LFP and Li|LFP full cells at 0.5 C under lean electrolyte and low N/P.

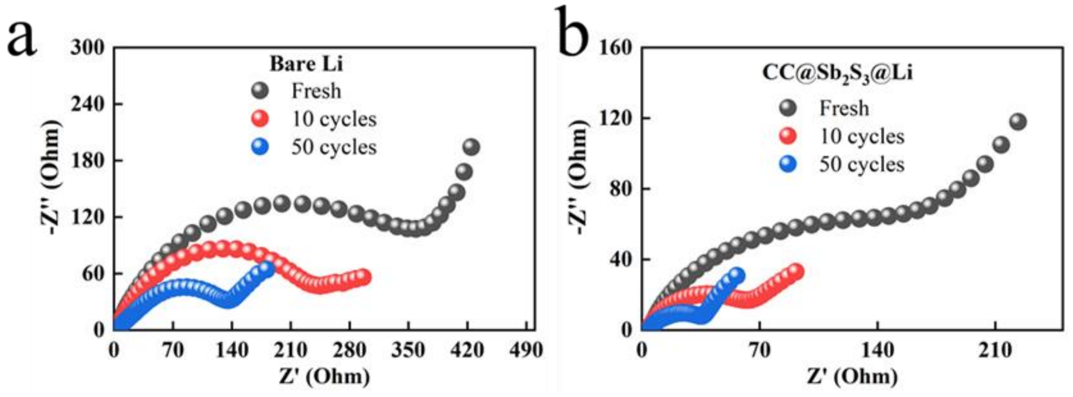

Figure S21. The cycling performance of full cells under more realistic conditions coupled with NCM523 cathode under lean electrolyte (4.4 µl mAh^{-1}) and low N/P (~0.44) at 0.5 C. The electrochemical impendence spectra of a) Li|NCM523 and b) CC@Sb2S3@Li|NCM523 cells after different cycles.

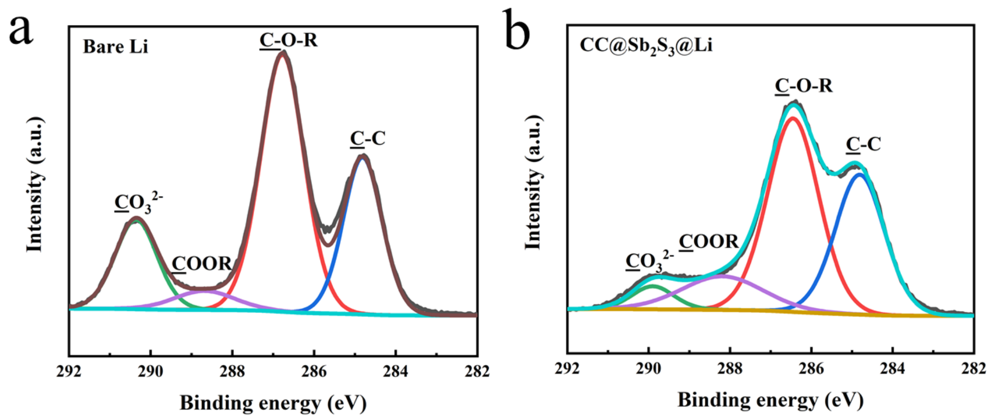

Figure S22. The XPS analysis of C 1s of different Li anodes in Li|NCM523 full cell after 50 cycles at 0.5 C.

Table S1. Comparison of electrochemical performances of symmetric cells with different 3D Li hosts.

Host	Current density ($mA\ cm^{-2}$)	Capacity ($mA\ h\ cm^{-2}$)	Time (h)	Overpotential (mV)
NPCC[1]	3	1	600	30
ZIF-67/Co/Cu[2]	1	1	1100	11
MOF-HCF[3]	1	1	1000	20
3D Zn/ZnO[4]	4	1	500	40
NCH@CF[5]	3	3	500	23
N-doped graphene[6]	1	1	1400	15
Cu@MC[7]	1	1	400	12
SMC[8]	0.5	1	1600	20
ZnO-MCNCF[9]	3	1	275	23
mPPy-GO[10]	5	1	400	22
CC@Sb_2S_3 (this work)	1	1	1750	12
	5	1	1273	18
	3	3	1090	18

Table S2. Impedance fitting results for different electrodes before and after cycling.

Anode	Li foil			$CC@Sb_2S_3@Li$		
Impedance (Ω)	R_b	R_{SEI}	R_{ct}	R_b	R_{SEI}	R_{ct}
Before cycling	3.4	212.7	49.3	2.7	13.5	3.5
After 10 cycles	4.5	3.6	16.6	3.68	2.7	1.2

Table S3. Comparison of electrochemical performances of Li|LFP cells.

**Anode**	**LFP loading ($mg\ cm^{-2}$)**	**Rate (C)**	**Cycles**	**Capacity after cycling ($mAh\ cm^{-2}$)**
Au/Cu/Li nanoscaffold[11]	5	1	200	126
CC-Zn-CMFs-Li[12]	4.5	1	200	132
MgF_2-GO-Li[13]	13	1	150	110
Li_3P@Cu@Li[14]	8.5	1	240	120
FCMS@Li[15]	9.4	0.5	250	149
CFF/Co-Li_2O@Li[16]	3.2	1	250	149
GN@Cu foam Li[17]	3	0.5	300	127
N-doped graphene–Li[18]	5	0.2	500	137
CNT@POF@Li[19]	7.6	1	100	99.2
Li/ACrCFs[20]	10	1	100	135
$CC@Sb_2S_3@Li$ (this work)	13.36	1	400	135
	13.36	0.5 (N/P=0.44, E/C=4.4)	180	136.8

Reference

[1] K. Li, Z. Hu, J. Ma, S. Chen, D. Mu, J. Zhang, *Adv. Mater.* **2019**, *31*, 1902399.

[2] J. Man, W. Liu, H. Zhang, K. Liu, Y. Cui, J. Yin, X. Wang, J. Sun, *J. Mater. Chem. A* **2021**, *9*, 13661.

[3] Z. J. Zheng, Q. Su, Q. Zhang, X. C. Hu, Y. X. Yin, R. Wen, H. Ye, Z. B. Wang, Y. G. Guo, *Nano Energy* **2019**, *64*, 103910.

[4] Q. Chen, Y. Yang, H. Zheng, Q. Xie, X. Yan, Y. Ma, L. Wang, D. L. Peng, *J. Mater. Chem. A* **2019**, *7*, 11683.

[5] C. Chen, J. Guan, N. W. Li, Y. Lu, D. Luan, C. H. Zhang, G. Cheng, L. Yu, X. W. Lou, *Adv. Mater.* **2021**, *33*, 2100608.

[6] G. Huang, J. Han, F. Zhang, Z. Wang, H. Kashani, K. Watanabe, M. Chen, *Adv. Mater.* **2019**, *31*, 1805334.

[7] J. Qian, S. Wang, Y. Li, M. Zhang, F. Wang, Y. Zhao, Q. Sun, L. Li, F. Wu, R. Chen, *Adv. Funct. Mater.* **2021**, *31*, 2006950.

[8] F. Zhang, X. Liu, M. Yang, X. Cao, X. Huang, Y. Tian, F. Zhang, H. Li, *Nano Energy* **2020**, *69*, 104443.

[9] R. Zhang, Y. Li, L. Qiao, D. Li, J. Deng, J. Zhou, L. Xie, Y. Hou, T. Wang, W. Tian, J. Cao, F. Cheng, B. Yang, K. Liang, P. Chen, B. Kong, *Energy Storage Mater.* **2021**, *37*, 123.

[10] H. Shi, J. Qin, K. Huang, P. Lu, C. (John) Zhang, Y. Dong, M. Ye, Z. Liu, and Z.-S. Wu, *Angew. Chem. Int. Ed.* **2020**, *29*, 12147.

[11] H. Lin, Z. Zhang, Y. Wang, X. L. Zhang, Z. Tie, Z. Jin, *Adv. Funct. Mater.* **2021**, *31*, 2102735.

[12] Y. Fang, Y. Zeng, Q. Jin, X. F. Lu, D. Luan, X. Zhang, X. W. Lou, *Angew. Chem. Int. Ed.* **2021**, *60*, 8515.

[13] Q. Xu, X. Yang, M. Rao, D. Lin, K. Yan, R. Du, J. Xu, Y. Zhang, D. Ye, S. Yang, G. Zhou, Y. Lu, Y. Qiu, *Energy Storage Mater.* **2020**, *26*, 73.

[14] Z. Luo, S. Li, L. Yang, Y. Tian, L. Xu, G. Zou, H. Hou, *Adv. Mater.* **2021**, *87*, 106212.

[15] C. Sun, T. Wu, J. Wang, W. Li, J. Jin, J. Yang, Z. Wen, *J. Mater. Chem. A* **2018**, *6*, 19159.

[16] F. Liu, Z. Jin, Z. Hu, Z. Zhang, W. Liu, Y. Yu, *Chem. An Asian J.* **2020**, *15*, 1057.

[17] G. Yang, J. Chen, P. Xiao, P. O. Agboola, I. Shakir, Y. Xu, *J. Mater. Chem. A* **2018**, *6*, 9899.

[18] G. Huang, J. Han, F. Zhang, Z. Wang, H. Kashani, K. Watanabe, M. Chen, *Adv. Mater.* **2019**, *31*, 1805334.

[19] X. R. Chen, B. Q. Li, C. Zhu, R. Zhang, X. B. Cheng, J. Q. Huang, Q. Zhang, *Adv. Energy Mater.* **2019**, *9*, 1901932.

[20] J. Xiao, N. Xiao, C. Liu, H. Li, X. Pan, X. Zhang, J. Bai, Z. Guo, X. Ma, J. Qiu, *Small* **2020**, *16*, 2003827.
